# Human versus Computer Controlled Selection of Ventilator Settings: An Evaluation of Adaptive Support Ventilation and Mid-Frequency Ventilation

**DOI:** 10.1155/2012/204314

**Published:** 2012-10-15

**Authors:** Eduardo Mireles-Cabodevila, Enrique Diaz-Guzman, Alejandro C. Arroliga, Robert L. Chatburn

**Affiliations:** ^1^Department of Pulmonary and Critical Care Medicine, University of Arkansas for Medical Sciences, 4301 West Markham Street, Slot 555, Little Rock, AR 77205, USA; ^2^Respiratory Institute, Cleveland Clinic, 9500 Euclid Avenue, A90, Cleveland, OH 44195, USA; ^3^Department of Pulmonary and Critical Care, University of Kentucky, Lexington, KY 40536-0284, USA; ^4^Department of Medicine, Scott and White and Texas A and M Health Science Center College of Medicine, 2401 South 31st Street, Temple, TX 76508, USA

## Abstract

*Background*. There are modes of mechanical ventilation that can select ventilator settings with computer controlled algorithms (targeting schemes). Two examples are adaptive support ventilation (ASV) and mid-frequency ventilation (MFV). We studied how different clinician-chosen ventilator settings are from these computer algorithms under different scenarios. *Methods*. A survey of critical care clinicians provided reference ventilator settings for a 70 kg paralyzed patient in five clinical/physiological scenarios. The survey-derived values for minute ventilation and minute alveolar ventilation were used as goals for ASV and MFV, respectively. A lung simulator programmed with each scenario's respiratory system characteristics was ventilated using the clinician, ASV, and MFV settings. *Results*. Tidal volumes ranged from 6.1 to 8.3 mL/kg for the clinician, 6.7 to 11.9 mL/kg for ASV, and 3.5 to 9.9 mL/kg for MFV. Inspiratory pressures were lower for ASV and MFV. Clinician-selected tidal volumes were similar to the ASV settings for all scenarios except for asthma, in which the tidal volumes were larger for ASV and MFV. MFV delivered the same alveolar minute ventilation with higher end expiratory and lower end inspiratory volumes. *Conclusions*. There are differences and similarities among initial ventilator settings selected by humans and computers for various clinical scenarios. The ventilation outcomes are the result of the lung physiological characteristics and their interaction with the targeting scheme.

## 1. Introduction

The evolution of the computerized control of mechanical ventilators has reached the level where the ventilator can select some (previously human selected) settings based on computer controlled targeting schemes [1–4]. One of these control algorithms is called an “optimum targeting scheme” for which the only commercially available mode is adaptive support ventilation (ASV). “Optimum”, in this context means to minimize the work rate of breathing a patient would have to do if breathing unassisted with the ventilator selected tidal volume and frequency [5, 6]. These settings are based on the ventilator's assessment of respiratory system characteristics (i.e., alveolar minute ventilation requirement, estimated dead space volume, and expiratory time constant). Although ASV has embedded rules that attempt to prevent hypoventilation, air trapping, and volutrauma, the primary goal is not the prevention of lung injury. ASV has been reported to choose ventilator settings that provide adequate ventilation in patients with a variety of clinical conditions [7–9].

We developed mid-frequency ventilation (MFV) [10], a mode of ventilation using an optimum targeting scheme with the goal of maximizing alveolar ventilation and minimizing tidal volume to promote lung protection. The theoretical basis for MFV has been described elsewhere [10]. In brief, MFV uses a mathematical model for pressure control ventilation where patient characteristics (alveolar minute ventilation requirement, dead space ratio, and inspiratory, and expiratory time constants) are used to calculate optimal frequency and tidal volume settings. In this case, optimum is defined as the frequency and tidal volume that produce the maximum alveolar minute ventilation for a given inspiratory pressure setting (above PEEP). MFV results in higher ventilator frequencies delivering the lowest tidal volume possible for a given target minute ventilation and inspiratory pressure, while using a conventional ventilator. 

In order to allow a computer to choose ventilator settings, the clinician, must trust the process by which these settings are determined. Although several studies have been published with ASV, [1, 8, 9, 11, 12] none compared them directly to human performance. The purpose of this study was to compare the initial ventilator settings selected by human operators with those selected by two computer algorithms (ASV and MFV) in five hypothetical clinical scenarios.

## 2. Materials and Methods

The study was divided into 2 steps. The first step was to determine the clinician-selected ventilator settings. A survey was made available to all the medical and surgical critical care physicians, fellows, and respiratory therapists at the Cleveland Clinic. The survey asked for proposed ventilator settings for five hypothetical patient scenarios. The second step was to evaluate the ventilation outcomes (tidal volume, lung volumes, and airway pressures). We used a lung simulator programmed with the scenarios' respiratory system characteristics ventilated with the clinician-selected settings, ASV, and MFV. 

### 2.1. First Step: Electronic Survey

The Institutional Review Board approved the survey. An electronic survey (http://www.surveymonkey.com/, Portland, OR) was sent by email to faculty and fellows and posted in the respiratory therapy website from December 1 to 31, 2007. The survey presented five clinical scenarios (Table 1). All the scenarios used the same baseline parameters: a 70 kg predicted body weight, male, paralyzed. The scenarios included a patient with normal lungs, two patients with restrictive disorders (ARDS and morbid obesity), and two with obstructive lung disease (COPD and status asthmaticus). The scenarios were hypothetical, and included arterial blood gases (validity confirmed by the Henderson-Hasselbalch formula [13]); ventilator settings in volume controlled continuous mandatory ventilation and previously published values for lung resistance and compliance for each condition (Table 1). The survey asked what ventilator settings the clinician would choose for each scenario. The options were tidal volume goal, respiratory rate, I : E ratio, and PEEP. 

We only used the results from surveys that had all answers completed. The survey results were used to calculate the clinician goal for minute ventilation (respiratory rate multiplied by tidal volume) and alveolar minute ventilation (tidal volume minus dead space volume (estimated as 2.2 mL/kg) multiplied by respiratory rate). A fixed dead space volume was used in all clinical scenarios to fully appreciate the effects of the settings.

### 2.2. Second Step: Lung Simulator

We used a lung simulator (Ingmar ASL 5000, IngMar Medical Ltd., Pittsburgh, PA) to recreate the clinical scenarios in the survey. The simulator was set up as a passive respiratory system composed of a single linear constant resistance and single constant compliance. The respiratory system compliances and resistances used in the survey were programmed for each clinical scenario (Table 1). The parameters were constant during the experiments. Data from the simulator were recorded in a high-resolution file (500 Hz sampling frequency). Tidal volumes and end inspiratory and end expiratory volumes were measured as the excursion of the piston inside the lung simulator. 

Two mechanical ventilators were used: a Hamilton Galileo, (Hamilton Medical AG, Bonaduz, Switzerland) to deliver clinician settings (with pressure control ventilation) and ASV and a Dräger Evita XL (Dräger Medical AG & Co., Lübeck, Germany) to deliver MFV. The change in ventilator to deliver the MFV was due to our previous experience [10] that showed the Dräger ventilator generating the sharply rectangular pressure waveform necessary for efficient MFV. The ventilators were connected to the lung simulator using a conventional circuit (70 inches long) with separate inspiratory and expiratory limbs (Airlife; Cardinal Health, McGaw Park, IL) without a humidifier chamber. All experiments were conducted using room air (F_I_O_2_ = 0.21) and reported as measured. The ventilators were calibrated and tested for leaks prior to the experiments. 

### 2.3. Experimental Protocol

#### 2.3.1. Clinician Settings

For each of the clinical scenarios we obtained the average tidal volume, respiratory rate, I : E ratio, and PEEP selected in the survey. The ventilator was set with these values.

To maintain comparability with MFV and ASV (both pressure controlled modes), clinician ventilator settings were delivered with pressure controlled continuous mandatory ventilation (i.e., all breaths were time triggered, pressure limited, and time cycled). Inspiratory pressure was determined in a preliminary run on the simulator to achieve the target tidal volume. Pressure rise time was set to the minimum available on each ventilator (Hamilton 50 ms, Dräger 0 ms). 

#### 2.3.2. Adaptive Support Ventilation

The ventilator was programmed for ventilation on an adult male patient. The height was set at 174 cm, which represents 70 kg of predicted body weight. For each case scenario, the percent minute ventilation was set to achieve the target minute ventilation obtained from the clinician survey. ASV was maintained until ventilation parameters were stable (no change in tidal volume, respiratory rate or inspiratory pressure).

#### 2.3.3. Mid-Frequency Ventilation

MFV uses pressure control continuous mandatory ventilation, with constant I : E ratio as frequency is changed. The ventilation parameters obtained from the computerized model for MFV [10] were used to program the ventilator to achieve the target alveolar ventilation.

**Figure 1:**
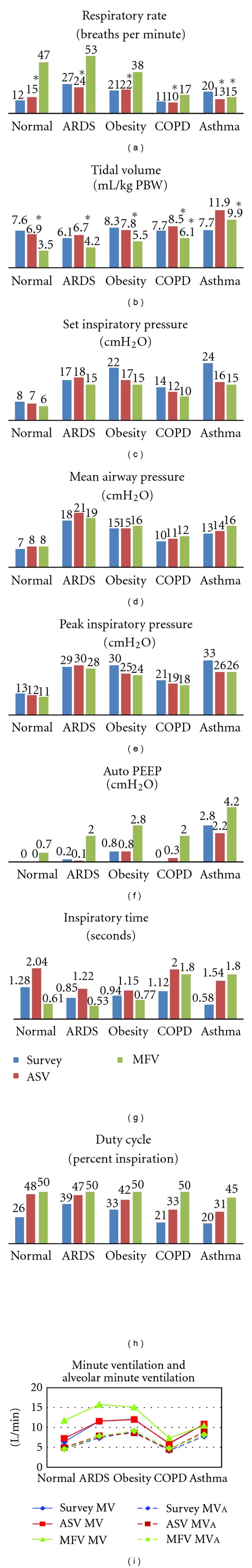
Clinician-selected settings, adaptive support ventilation and mid-frequency ventilation applied to lung simulator. (a) Respiratory rate; (b) tidal volume as registered by the lung simulator; (c) set inspiratory pressure above PEEP needed to deliver target tidal volume; (d) mean airway pressure; (e) peak inspiratory pressure; (f) auto PEEP; (g) inspiratory time; (h) duty cycle or percent time in inspiration; (i) exhaled minute ventilation (MV) and calculated alveolar minute ventilation (MV_A_). Values measured by the lung simulator: (b, d, e, g, h, and i). *Value within the range obtained from the clinician survey.

The target minute ventilation used to set ASV and target alveolar ventilation used to set MFV are shown in Table 2. The values for initial ventilator settings for MFV are reported in Figure 1.

#### 2.3.4. PEEP

Note that the survey asked for PEEP values, and these were used to set the ventilator and the model. However, contrary to clinical practice, PEEP has no effect on the lung simulator's behavior. More to the point, none of the computerized systems calculates or sets PEEP. Hence, the comparison between human and computer selection of ventilator settings is focused on frequency and tidal volume and ventilation outcomes.

## 3. Statistical Analysis

Results were analyzed with JMP IN (SAS, Cary, NC). The survey results are reported as mean and standard deviation. The results and respective comparisons between groups on the lung model are only descriptive. The lung model generates values with virtually zero standard deviation; hence, only descriptive statistics are reported.

## 4. Results

### 4.1. Clinician Settings Survey Results

The electronic survey was available to 176 respiratory therapist, fellows, and staff from the medical, surgical, and cardiothoracic intensive care units of the Cleveland Clinic. A total of 54 surveys were collected at the end of the study period. Of these, only 33 were completely answered (13 (39%) critical care staff, 6 (18%) critical care fellows, and 14 (42%) intensive care respiratory therapist) and were used to obtain the reference values.

Table 2 depicts the results of the survey and the calculated minute ventilation and alveolar ventilation. Tidal volumes ranged from 6.1 mL/kg (ARDS) to 8.3 mL/kg (morbid obesity). Clinicians reduced tidal volumes (range 0.9 to 2.3 mL/kg) from the tidal volume used in the scenario. In the obstructive lung disease scenarios, clinicians decreased respiratory rate on average 1 to 2 breaths per minute. 

The minute ventilation calculated from survey response was in accordance with the acid-base disorder (i.e., an increase in minute ventilation for acidosis and a decrease for respiratory alkalosis). The clinicians' selected minute ventilation (6.4 L/min) was similar to the calculated normal minute ventilation (7.0 L/min) for a healthy patient >15 kg (i.e., 100 mL/kg/min ideal body weight) [5]. 

### 4.2. Survey Results, Adaptive Support Ventilation, and Mid-Frequency Ventilation Applied on to a Lung Simulator

Figure 1 shows the results of the survey, ASV, and MFV when applied to the lung simulator. The tidal volume selected was 6.7 to 11.9 mL/kg for ASV, and 3.5 to 9.9 mL/kg for MFV. The difference between ASV and the clinician-selected tidal volumes was negligible (−0.9 to 0.7 mL/kg), with the exception of status asthmaticus where tidal volume selected by ASV was 3.9 mL/kg (55%) larger. MFV selected tidal volumes that were 1.5 to 4.1 mL/kg lower than the clinician-selected tidal volumes, with the exception of asthma, were MFV selected tidal volumes were larger (2.2 mL/kg). MFV used higher respiratory rates and lower tidal volumes. This was most evident in normal and restrictive physiology. In obstructive scenarios, both ASV and MFV used lower respiratory rates (in status asthmaticus even lower than the clinicians) combined with longer inspiratory times (a result of larger duty cycles).

Figure 1(i) compares the calculated minute ventilation with the one delivered by MFV and ASV. Of note is how calculated alveolar ventilation goals were equal with each mode.

**Figure 2:**
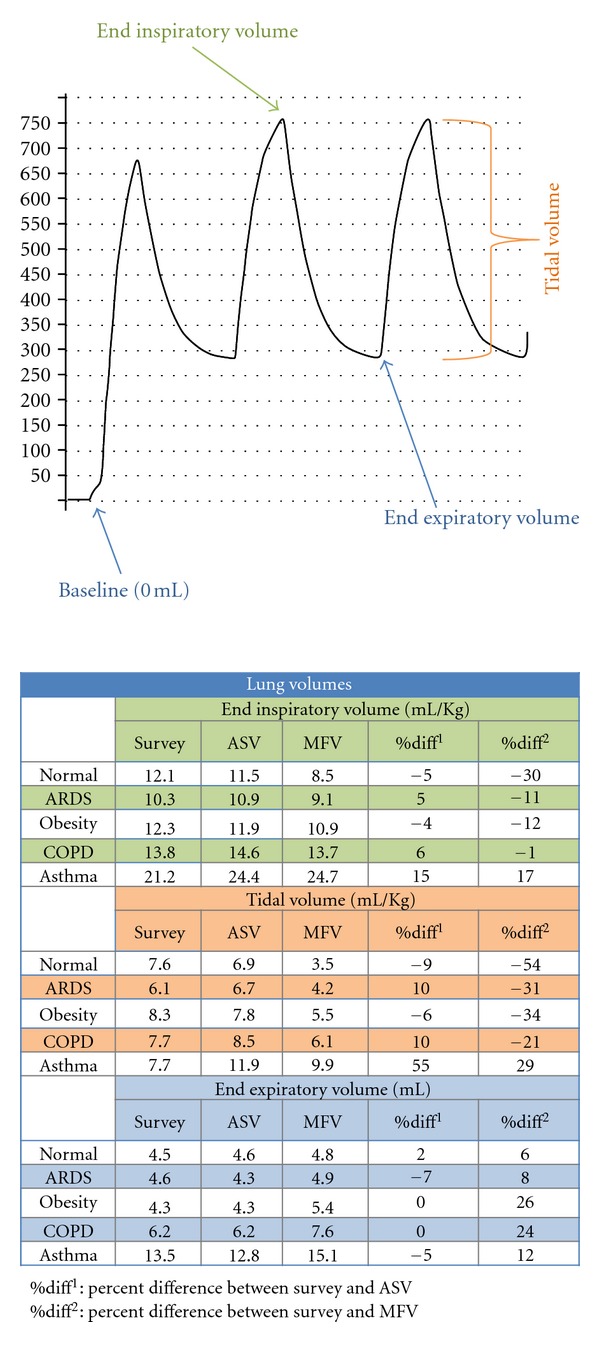
Lung volumes and ventilator settings. End inspiratory and expiratory volumes are total volumes normalized by weight as measured from the baseline. Baseline is 0 mL in the lung simulator; hence, end expiratory volume is a reflection of PEEP and aPEEP and the end inspiratory volume is a manifestation of PEEP, aPEEP, and tidal volume. Tidal volume was measured as the excursion of the simulator piston; however, it can also be estimated by subtracting the end expiratory from the end inspiratory volumes.

Figure 2 depicts the effects and differences in lung volumes between ventilator settings. With the exception of asthma, ASV had very similar (within 6%) end inspiratory volumes (EIV) and end expiratory volumes (EEV) to the clinician survey. MFV had 6–12% larger EEV, but when coupled with lower tidal volumes resulted in 1–30% less EIV. In the asthma scenario, both computer algorithms had larger EIV (15% ASV and 17% MFV) than the clinicians.

Mean airway pressures (mP_AW_) were similar for all strategies (within 3 cm H_2_O), with a trend towards higher values in MFV (probably due to the more rectangular pressure waveform of the Dräger ventilator compared to the Hamilton ventilator). Peak inspiratory pressure tended to be lower with the computer algorithms; this difference was small (within 3 cm H_2_O) with the exception of obesity and asthma where the difference was more evident (*≈*6 cm H_2_O). MFV used less inspiratory pressure for all scenarios than ASV and the clinician settings. AutoPEEP (aPEEP) was identical between ASV and the clinician-selected values and was essentially nonexistent with the exception of asthma. MFV had consistently higher (*≈*2 cm H_2_O) aPEEP than the other strategies. The duty cycle was higher in MFV and ASV for all scenarios; however, because of the high respiratory rate in restrictive and normal lungs the inspiratory times were usually shorter for MFV.

## 5. Discussion

Our study demonstrates the differences between the clinician's settings and closed loop targeting schemes. These differences may not be significant in some cases. For example, ASV yields ventilation settings and ventilation outcomes very similar to clinician's choice and published guidelines for scenarios as ARDS and normal lungs but not for obstructive disorders. While, MFV results in less volume (both tidal volume and end inspiratory volume) and pressure (for most scenarios) than either ASV or clinicians. 

ASV and MFV are examples of optimal control targeting schemes for mechanical ventilators [18]. Yet the modes have different optimization goals: ASV's optimum settings aim to minimize the work rate of breathing, while MFV's goal is to maximize alveolar ventilation and minimize tidal volume. MFV is not currently available as a mode on ventilators, so there are no published studies of clinical outcomes. Studies have evaluated ASV as the sole mode of ventilation [9], or in patients with stable gas exchange (without reporting baseline ventilator settings) [8], or had a specific protocols to set the comparator ventilator settings (fixed tidal volume, SIMV) regardless of lung disease or mechanics [19–21] or where done with ASV prototypes [1, 11]. Our study eliminated variability by utilizing the minute ventilation goals chosen by clinicians to set two optimal control modes. These yielded information on the effects of current ventilation strategies and those of computerized models.

In normal lung physiology, ASV chosen ventilator settings were similar to the clinician's choice and tidal volume (6.9 mL/kg) was within the range considered to be lung protective. MFV used 54% less tidal volume (3.5 mL/kg) which was associated with a 30% reduction in end inspiratory volume. Interestingly, there was minimal difference in aPEEP, PIP, and mP_AW_ amongst the three settings. 

 In ARDS, the tidal volume used by ASV was 0.6 mL/kg (10%) higher than the clinician's choice, well within range considered to be lung protective [22, 23]. The ASV algorithm uses >1 respiratory time constant to set the inspiratory time [5] which led to longer inspiratory times and thus contributed to a higher mP_AW_ compared to the clinicians. In the obesity scenario, where compliance and resistance were higher, ASV used lower tidal volume (0.5 mL/kg) with slightly longer inspiratory time resulting in the similar airway pressure. In comparison, in both restrictive disorders, MFV used higher than normal respiratory rates to deliver 31–34% lower tidal volumes than clinicians, resulting in an EEV 8–26% higher (recruitment) and 11-12% lower EIV (stretching). The combination of low EIV and high EEV, especially in restrictive lung disorders, are in concordance with MFV goal to maximize lung protection, that is, preventing atelectrauma and alveolar stretching. 

In obstructive disorders, clinicians used different patterns of ventilation for COPD (low tidal volume/low respiratory rate) and status asthmaticus (low tidal volume/high respiratory rate) while the computerized models used the same pattern (large tidal volume/low respiratory rates) for both scenarios. Our results are in concordance with the ventilator setting patterns found by Arnal et al. [9] and Belliato et al. [8] in COPD (they did not report patients with status asthmaticus). The discrepancy in the clinician's ventilation pattern choice for obstructive disease can be explained by three situations. First, the COPD scenario depicted a patient with respiratory alkalosis due to overventilation, which intuitively required less minute ventilation, compared with severe respiratory acidosis in status asthmaticus (requiring an improvement not only in MV but also in gas exchange). Second, clinicians are used to managing respiratory failure due to COPD, not status asthmaticus. The reduction in cases of status asthmaticus requiring mechanical ventilation [24] may have led clinicians to become less familiar with the management of this condition. The goal of ventilator management in status asthmatics has been to prolong the expiratory phase (i.e., decreasing the I : E by low respiratory rate, high flows, and short inspiratory time) while tolerating hypercapnia and acidosis [25, 26]. This “lack of practice” may also explain the high level of PEEP selected in a paralyzed patient where no trigger asynchrony could occur and where, although controversial, it could worsen air trapping [27, 28]. Lastly, low tidal volume ventilation is being applied to everyone [29]. Although the trend in patient with status asthmaticus [24] was present prior to the ARDS net seminal article [23] it was likely enhanced by it. As a matter of fact, the tidal volume recommended in review articles through time has decreased (1980's: 10–12 [26, 30] 1990's: 8–10 [26, 31] 2000's: 8–10 [32], 6–8 [28, 33] 5–7 [34, 35] mL/kg) in the absence of any new clinical observations since those of Darioli and Perret [25] and Tuxen et al. [26, 36].

 There are limitations to our study. First, the survey sampled critical care physicians and respiratory therapists in a single large academic institution. The poor response rate may have been due to the time of the year (December holidays) and inadequate delivery of the survey (i.e., to some departments the survey was made available through a website rather than direct email). While recognizing that the sample is small and obtained during a holiday period, the objective of the survey was to obtain a measure of how clinicians react to ventilation scenarios. The survey may not represent regional or national practices; however, it represents a snap shot of mechanical ventilation in a large academic institution during a given moment in time. It can be argued that “expert” clinicians would do better than what the survey revealed, or that guidelines indicate different courses of action. We concede that setting the ventilator is a complex process where changes to parameters should be made in response to airway pressure measurements and clinical findings. Further, the initial settings are changed according to response, sometimes immediately. Yet, the survey results do demonstrate that clinicians sometimes fail to follow guidelines, protocols, or physiology. “Expert” clinicians are not available at the bedside all the time. The variability in settings chosen by clinicians, some against common teachings, is the strength of the study. This variablity represents differences in humans experience, levels of education, and propensity to follow protocols. For better or worse, computers assure adherence to protocols.

Another limitation is the reliance on a relatively simple version of the equation of motion as the basis for the mathematical models used by the computer algorithms and the lung simulator. The equation considers the lung as a single alveolar unit with constant compliance and resistance, which is an oversimplification of the heterogeneous nature of the lung, particularly in disease states. However, two factors support its application in this study. First, the equation of motion has been used in commercially available ventilator targeting schemes (Proportional Assist Ventilation Plus, Proportional Pressure Support, and Adaptive Support Ventilation) [9, 37]. Second, and most important to our study, Belliato et al. [8] demonstrated that in passive conditions, the lung simulator we used, when programmed with the measured patient lung resistance and compliance behaved identically to the patients studied (same pressures, and volumes). The simulator allowed us to obtain data which would have been impossible to obtain in “real life conditions” since the same patients could not be sensibly placed on three ventilator settings without changing the clinical and respiratory system status. 

Another limitation is the lack of respiratory effort during this study. The absence of respiratory effort in clinical practice is the exception rather than the rule. For example, Arnal et al. [9] showed that in spontaneously breathing patients, the ventilator settings chosen by ASV were similar regardless of physiology, and were only different in extreme restrictive and obstructive lung disease. ASV uses adaptive pressure targeting in spontaneously breathing patients. That is, the patient decides the tidal volume and respiratory rate, thus the observed breathing pattern is less dependent on the ventilator settings and more dependent on the patient respiratory drive. It is still to be determined what the behavior and role of MFV would be in spontaneously breathing patients.

 Finally, the fact that ASV and MFV use a closed loop control to find the settings to achieve the target minute ventilation means that the initial settings chosen by the device are adjusted over the next minutes to achieve the target goal. This would inherently bias the results towards ASV and MFV, as the clinician did not have a chance to optimize its settings based on ventilation outcomes. However, the goal of the study was to demonstrate the differences in choices, and given that this was a static model, the settings chosen by the closed loop algorithms had minimal variation.

## 6. Conclusions

Computer controlled targeting schemes may result in similar ventilator settings to those chosen by a clinician (e.g., ASV in normal lung physiology) or very different settings (e.g., MFV in ARDS physiology delivering less volume and pressure) for the same minute ventilation goal. The targeting scheme's goal and its interaction with the lung physiological characteristics explain these differences. 

## Figures and Tables

**Table 1:** Clinical scenarios.

Clinical Scenario	Acid-base and oxygenation status	Compliance mL/cm H_2_O	Resistance cm H_2_O/L/S
*Healthy patient* undergoes surgical repair of the knee. He has a rare enzymatic defect that prolonged the paralytic half-life and will require mechanical ventilation until paralysis wears off.	*Normal acid-base status*. ABG: pH 7.40, PaCO_2_ 40, HCO_3_ of 24, and PaO_2_ 90 on a 30% FiO_2_	**66**	**4**

*ARDS* due to severe sepsis. Current ventilation: VC-CMV, V_T_ 550, RR 28, FiO_2_ 65%, PEEP of 12.	*Metabolic and respiratory acidosis.* ABG: pH 7.25, PaCO_2_ 42, HCO_3_ of 18 and PaO_2_ 65 on a 65% FiO_2_	**25**	**10**

*Morbid obesity*: weight is 200 kg, he has opiate overdose. He is paralyzed due to high ventilator pressures. Current ventilation: VC-CMV, V_T_ 650, RR 18, FiO_2_ 35%, PEEP of 8.	*Respiratory acidosis.* ABG: pH 7.27, PaCO_2_ 85, HCO_3 _of 38 and PaO_2_ 65 on a 35% FiO_2_.	**35**	**12**

*COPD*: has a broken hip, intubated for surgery. Current ventilation: VC-CMV, V_T_ 700, RR 12, FiO_2_ 35%, PEEP of 8, and I : E 1 : 4.	*Respiratory alkalosis.* ABG: pH 7.53, PaCO_2_ 42, HCO_3_ of 34 and PaO_2_ 65. No auto PEEP is detected.	**60**	**16**

*Status asthmaticus*: paralyzed in the ED to facilitate ventilation. Current ventilation: VC-CMV, V_T_ 600, RR 22, FiO_2_ 35%, PEEP of 10, I : E is 1 : 2.	*Severe respiratory acidosis.* ABG: pH 7.12, PaCO_2_ 75, HCO_3_ of 24 and PaO_2_ 65 on a 35% FiO_2_. Auto PEEP is 6.	**80**	**Inspiratory 16** **Expiratory 22**

VC-CMV: Volume control-continuous mandatory ventilation, V_T_: tidal volume, RR: respiratory rate (breaths per minute), I : E: inspiratory : expiratory ratio, PEEP: positive end expiratory pressure (cm H_2_O), ABG: arterial blood gas. PaCO_2_: arterial partial pressure of CO_2_ (mmHg), PaO_2_: arterial partial pressure of O_2_ (mmHg), FiO_2_: inspired fraction O_2_. References: healthy paralyzed [14], ARDS [15, 16], morbid obesity [14], COPD [15], status asthmaticus [17].

**Table 2:** Survey results.

Condition	Tidal volume mL	Tidal volume mL/kg (*)	Alveolar volume mL	RR bpm (*)	PEEP cm H_2_O	I : E ratio (DC)	MV calc L/min	MV_A_ calc L/min
Normal lungs/normal acid-base	535 ± 89	7.6 (5.7–10)	382	12 ± 3 (8–23)	5 ± 1	1 : 3 (25)	6.4	4.6
ARDS/mixed acidosis	428 ± 38	6.1 (5–7.1)	275	27 ± 7 (10–42)	12 ± 2	1 : 1.5 (40)	11.6	7.4
Obesity/respiratory acidosis	578 ± 105	8.3 (5.7–11.4)	425	21 ± 3 (12–30)	8 ± 2	1 : 2 (33)	12.1	8.9
COPD/respiratory alkalosis	536 ± 80	7.7 (5.7–10)	383	11 ± 2 (6–16)	7 ± 2	1 : 4 (20)	5.9	4.2
S. asthmaticus/respiratory acidosis	542 ± 102	7.7 (5.7–11.4)	389	20 ± 6 (6–30)	9 ± 4	1 : 4 (20)	10.8	7.8

Values are expressed as mean ± SD or mean alone. (*): range. Tidal volume per kg of predicted body weight (70 kg), alveolar volume was calculated by subtracting 153 mL (2.2 mL/Kg) dead space volume from the average tidal volume. BPM: breaths per minute. DC: duty cycle or percent inspiration. PC mode choice includes adaptive PC, PC-CMV, and IMV. MV: minute ventilation. MV_A_: alveolar minute ventilation. PEEP: positive end expiratory pressure.
